# Patient and Family Involvement in Nursing Bedside Handover: A Qualitative Descriptive Study of Consumer Perceptions of Nursing Care

**DOI:** 10.3390/nursrep15020051

**Published:** 2025-02-03

**Authors:** Manonita Ghosh, Beverly O’Connell, Hien Thi Nguyen, Linda Coventry, Amanda Towell-Barnard, Olivia Gallagher, Karen Gullick, Lucy Gent, Rosemary Saunders

**Affiliations:** 1 Social Ageing Future Lab, School of Arts and Humanities, 270 Joondalup Drive, Joondalup 6027, Australia; thihien.nguyen@ecu.edu.au; 2 School of Nursing and Midwifery, Edith Cowan University, 270 Joondalup Drive, Joondalup 6027, Australia; b.oconnell@ecu.edu.au (B.O.); l.coventry@ecu.edu.au (L.C.); a.towell-barnard@ecu.edu.au (A.T.-B.); lucy.gent@health.wa.gov.au (L.G.); rosemary.saunders@ecu.edu.au (R.S.); 3 Centre for Research in Aged Care, Edith Cowan University, 270 Joondalup Drive, Joondalup 6027, Australia; 4 Centre for Nursing Research, Sir Charles Gairdner and Osborne Park Health Care Group, Nedlands 6009, Australia; 5 School of Allied Health, University of Western Australia, Nedlands 6009, Australia; olivia.gallagher@uwa.edu.au; 6 Hollywood Private Hospital, Monash Avenue, Nedlands 6009, Australia; gullickk@ramsayhealth.com.au

**Keywords:** nursing, bedside handover, nurse–patient interaction, patient–family-centred care, patient and family involvement, patient satisfaction, patient and family perspectives, qualitative research

## Abstract

**Background/Objectives:** Patient and family involvement in bedside handover is a requirement of the national standards on patient safety and quality in Australia. To ensure patient-and-family-centred care, it is essential to understand how patients and families perceive their involvement in nursing bedside handover and what difficulties they face when participating. This study aimed to explore patient and family perceptions of their involvement in nursing bedside handover. **Methods:** We employed a qualitative descriptive study design with in-depth and semi-structured interviews. Using purposive and convenience sampling, 24 patients and family members were recruited from two adult hospitals in Western Australia between November 2021 and February 2022. The data were thematically analysed. **Results:** Participants had mixed experiences that overlapped with their individual perceptions, needs, and experiences. Their responses were grouped into three major themes with sub-themes: (1) discovering new nursing care approaches; (2) seeing the value of involvement in bedside handover; and (3) barriers hindering patient and family involvement in bedside handover. The findings revealed that patients and families valued their involvement in nursing bedside handover. However, several factors challenged their participation, including a lack of awareness about their right to participate, the timing of handovers, the nurse’s approach, and fear of asking questions. **Conclusions:** The findings serve as a guide for evidence-based practice and may significantly influence policy and practice in nursing bedside handover, potentially enhancing patient-and-family-centred care. While considered best practice, the consistent involvement of patients and their families in nursing bedside handover is not routinely achieved and is implemented to varying extents.

## 1. Introduction

Nursing bedside handover has gained increased attention since the 2000s, particularly after the World Health Organisation recognised it as a crucial strategy for enhancing safe patient care [1]. This practice is to be conducted by nurses at the patient’s bedside. The aim of nursing bedside handover is to share, confirm, and clarify clinical information and the patient’s current condition by engaging both the patient and their family during shift transitions. Effective bedside handovers involve clear communication of the patient’s status and treatment plan to nursing staff, patients, and their families, which is critical for ensuring continuity of care and promoting patient safety [1,2]. To ensure an effective nursing bedside handover process, Chaboyer et al. [3] recommended five steps: First, prepare by allocating staff and patients and updating patient information and care documents. Second, outgoing nurses introduce the oncoming staff and patients. Third, exchange information through face-to-face communication, patient care records, and staff questions. Fourth, involve patients by inviting them to comment or ask questions to enhance the quality and accuracy of the handover. Finally, oncoming nurses perform a safety check of patients’ conditions, environment, and equipment.

The Australian Commission on Safety and Quality in Health Care has highlighted the importance of effective communication between healthcare professionals and patients and their families [4]. For the past decade, bedside handovers have been mandated in all Australian hospitals as part of accreditation requirements. This requirement is to guarantee that patients and their families are involved in the handover process, ensuring the safe delivery of healthcare and fostering patient-and-family-centred care [5]. However, simply conducting bedside handovers does not guarantee active patient and family involvement or effective communication among nurses, patients, and their families. There is limited current evidence on whether patients and families are actively participating in nursing bedside handovers and whether their perceptions are being considered.

Numerous studies have been conducted on the topic of bedside handover, primarily focusing on the perceptions of nurses [6,7,8]. However, there is a paucity of research exploring patient perceptions of bedside nursing handover. A systematic review reported that patients’ participation was mostly influenced by nurses, with patients being more inclined to participate when nurses made deliberate attempts to include them in the dialogue [9]. Another systematic review found that nurses often dominated the conversation during the bedside handover, leading patients to feel ignored [10]. Mullen et al. [11] explored the perceptions of mental health patients regarding their involvement in nursing handover at a regional hospital in Australia. They reported a lack of interaction between patients and nurses, with patients feeling “shunned or ignored” during handover [11] (p. 732). Similarly, Anshasi and Almayasi [12] reported that patients felt excluded or neglected by nurses during bedside handover, largely due to the use of medical jargon and insufficient attention to the fact that the patient was listening and not involving the patients in the conversation.

In a systematic review, aimed at gaining a deeper understanding of patient experiences in bedside handover, Bressan et al. [13] recognised that understanding patients’ perceptions and experiences is crucial for designing and implementing a process that respectfully incorporates the preferences and values of both patients and their families. They reported that patient involvement in bedside handover did not occur until 2013. Additionally, they noted that studies examining patient perspectives were mainly conducted in medical and surgical units and in single hospitals. They suggested that further studies involving patients with different clinical conditions, at different stages of life, and with varying lengths of hospital stay are needed.

To address the gap, we conducted a mixed-methods study to explore patient and family perceptions of their involvement in nursing bedside handover in two Western Australian (WA) hospitals involving all wards and units including medical, surgical, critical, and mental with a longer hospital stay. The mixed-method study included a cross-sectional survey incorporating both closed- and open-ended questions, followed by individual semi-structured interviews using a qualitative descriptive approach to provide deeper insights into consumer perceptions of nursing care [14]. The findings from the cross-sectional survey, including the triangulated qualitative content analysis, have been reported in a previous study [15]. In this paper, we report the findings from the interviews guided by two key research questions: How did patients and family perceive their involvement in bedside handover? What promoted or challenged their involvement in the handover?

## 2. Methods

### 2.1. Study Design

In this current study, we employed a qualitative descriptive design to address the paucity of research on patient and family perceptions of their involvement in nursing bedside handovers, as it offers clear, straightforward, and rich descriptions of individuals’ perceptions and experiences [16]. The qualitative descriptive approach is commonly used in nursing and healthcare research, often serving as the qualitative component in mixed-methods studies due to its simplicity, flexibility, utility, and time efficiency [17,18]. This methodology shares the core philosophical foundation of qualitative research while also incorporating unique features such as conducting the study in a naturalistic setting, gathering data that reflect participants’ experiences, and presenting findings consistent with the data provided by participants [17,18]. This study was also guided by the Standards for Reporting Qualitative Research (SRQR) Framework [19] (Supplementary Material).

### 2.2. Settings and Participants

This study was conducted in two adult hospitals in WA, one public and one private. The public hospital, one of the largest teaching hospitals in WA, has 787 licensed beds and over 3000 nursing staff, caring for more than 420,000 patients annually. The private hospital, the largest in Australia, has 860 licensed beds and over 1300 nursing staff, caring for over 75,000 patients each year. All ward types, including medical, surgical, critical, and mental wards, in both hospitals were involved in this study. Nursing handover occurs at both hospitals during every shift change, and bedside handover occurs at least once per day. The specific times of bedside handover vary between the wards and units of each hospital. During the study period, all nurses working in the public hospital used the iSoBAR framework (Identification, Situation, Observation, Background, Assessment, and Recommendation) for bedside handover, and nurses in the private hospital used the SHARED framework (Situation, History, Assessment, Risk, Expectation, and Documentation) to guide patient handover.

We used a combination of purposive and convenience sampling, consistent with a qualitative descriptive approach [17,18]. Purposive sampling ensured the inclusion of participants who could provide the most valuable insights, enhancing the quality and relevance of the study findings. Participants included patients aged 18 and above who were admitted to any of the participating hospitals during a period when at least one bedside handover occurred, as well as their family members. Convenience sampling allowed us to select participants who were readily accessible and available.

Patients and families received a participant information form explaining the overall study and an invitation to complete a survey and an interview about their involvement in nursing bedside handover during their hospital stay. Participants were provided with a reply-paid envelope to return the survey about their involvement in nursing bedside handover during their hospital stay, and a form to record their contact details if they were interested in participating in a follow-up interview. Patients unable to provide informed consent were not eligible to participate.

In qualitative research, due to the focus on collecting rich and detailed information, sample sizes are typically small and not pre-determined. Data saturation is considered the gold standard for determining sample size [17,18]. A total of 93 individuals expressed their interest in participating in the follow-up qualitative interview. Of them, three had illegible contact or wrong numbers, seven withdrew, and another twelve did not respond to the phone call or email, leaving 71 prospective participants to be interviewed. Data saturation was achieved after conducting 24 interviews, at which point no new information emerged from the data.

### 2.3. Data Collection

One-on-one in-depth, semi-structured interviews were carried out by the first author between November 2021 and February 2022. Interviews were conducted over the phone through Microsoft Teams as this was convenient for the participants. The main components of the questions asked were (a) what role did they play in the bedside handover?; (b) how did their participation in the bedside handover impact care delivery?; (c) what challenges and barriers did they face in participating in the bedside handover?; and (d) how to improve the bedside handover to maximise their involvement in the bedside handover? Prompts were used to probe, clarify, and elicit deeper explanations from the participants. Demographic data collected were gender, age, relationship (patient/family), country of birth, level of education, employment status, number of admissions in the participating hospitals, and length of hospital stay. Each interview started by introducing the researcher and participant to each other; describing the purpose of the study, confidentiality, and consent; and ensuring the participant’s understanding of nursing bedside handover. The interview lasted for approximately 30–45 min. During the interview, participants agreed to be audio recorded, and information was kept confidential. Microsoft Teams Automatic Speech Recognition recorded and transcribed the real-time conversation into text.

### 2.4. Data Analysis

A step-by-step process was employed to conduct a thematic analysis, converting the raw data into distinct themes and sub-themes. The major steps were familiarising ourselves with the data, generating initial codes, searching for themes, reviewing the identified themes, and writing the manuscript [20]. We read each transcript several times to generate initial codes in which we labelled and organised the text data into groups to identify the underlying themes in the data. These codes included phrases or words which were of relevance or importance. We then reviewed the codes further to form main themes and sub-themes. A certain level of interpretation was inevitable in this process; however, we made efforts with the research team to closely align with the participants’ own expressions when identifying themes and sub-themes. The objective was to derive an overarching or abstracted meaning from the data that reflected key information about participants’ experiences. We selected appropriate and compelling quotations as examples, linking the analysis back to the research questions and existing literature.

### 2.5. Rigour

The trustworthiness and quality of our study were ensured through key criteria: credibility, confirmability, dependability, and transferability [18,21]. Credibility was derived from open-ended interviews, which provided in-depth insights and a thorough exploration of the experiences of patients and family members [21]. We ensured confirmability by providing detailed identification of participant characteristics, context, and thick descriptions [18]. Dependability was achieved through peer examination, where we familiarised ourselves with and continually immersed ourselves in the data, revising and checking the meanings, codes, themes, and sub-themes [21]. To confirm transferability, we employed purposeful sampling and included a detailed description of the interview guide in the data collection process [18].

### 2.6. Ethical Considerations

This study adhered to ethical principles according to the National Health and Medical Research Council National Statement on Ethical Conduct in Human Research [22]. Prior to the interview, written informed consent was obtained from all participants who were fully informed of the study’s purpose, objectives, and procedures. They were aware of their voluntary participation and their right to pause the audio recording or withdraw from the study at any time without giving an explanation. Assurance of data protection and confidentiality were provided. No identifiable information was included in the reports or publications. Furthermore, ethical approvals were rigorously obtained from the Human Research Ethics Committees of both participating hospitals (Department of Health-RGS0000004922, 9 September 2021, and Ramsay GEKO-34656 HREC 2107W, 14 May 2021), as well as Edith Cowan University (ECU-2021-02916-GHOSH, 21 September 2021).

## 3. Findings

**Figure 1:**
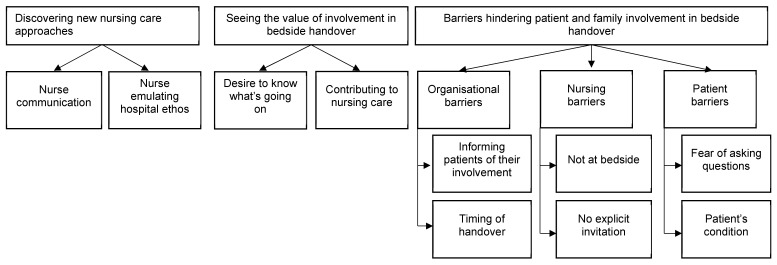
Themes and sub-themes of patient and family involvement in bedside handover.

There were 24 patients and families who participated in this study. Among them, 20 were patients and 4 were family members, with over half being female and born in Australia (Table 1). Their perceptions are presented in three major themes with 10 sub-themes, as shown in Figure 1. These themes and sub-themes are elucidated using verbatim extracts drawn from the participants’ narratives.

### 3.1. Discovering New Nursing Care Approaches

All participants observed bedside handover during their current admission but had not experienced it in previous admissions. This theme highlights how patients recognised the benefits of bedside handover through enhanced nurse communication and the embodiment of the hospital’s ethos. Patients perceived that improved nurse communication enhanced the quality of care they received, resulting in more personalised and responsive treatment. Patients noted that bedside handover allowed nurses to communicate more effectively, ensuring that important information was accurately conveyed and understood. This improved communication not only helped in addressing patients’ immediate needs but also in anticipating potential issues, thereby potentially preventing complications. The transparency and involvement in their own care plans made patients feel more informed and empowered.

Furthermore, by integrating the hospital’s core values and practices into the bedside handover process, nurses were able to demonstrate a commitment to high standards of care. This approach reinforced the hospital’s dedication to patient-centred care, where the needs and preferences of patients are prioritised. Patients felt that this alignment with the hospital’s ethos contributed to a more cohesive and supportive healthcare environment. As a result, patients reported higher levels of satisfaction with the healthcare services provided to them. They appreciated the attentiveness and professionalism of the nursing staff, which fostered a sense of trust and confidence in the care they received. Overall, the implementation of bedside handover was seen as a positive change that enhanced the overall patient experience.

#### 3.1.1. Nurse Communication

Patients perceived the bedside handover process as a structural improvement to enhance nurse–patient communication. They noticed a consistent occurrence of handover between nurses who kept them informed of their treatment without necessitating inquiries on their part. Patients who had a hospital stay a long time ago reflected on the changes they noticed, specifically regarding patient involvement in the handover process.

*… I thought the communication is a lot better. … I like to be kept informed and up to date on what was happening with my treatment. And in this visit, I felt much better than in the past [when] I had to ask, and this time the nurses … came and told me, though without asking I thought was, much, much better. … I was kept informed [and] … standard improvement … this is what pleased me the most.*

(Patient 1)

*… I thought that their handover, discharge procedure was in every way superior compared to the one we had in (name of another hospital). … the behaviour the nurses had here, … they were very good. I was able to talk with the nurse, and understand [sic] what they had done, what drugs they’d given.*

(Patient’s husband 12)

Despite noting improvement in the bedside handover process, patients felt that nurses did not have a comprehensive understanding of medical diagnoses, leading to unclear explanations to patients. They perceived communication gaps between nurses and doctors, making it difficult for nurses to respond to patient queries effectively. As a result, nurses advised patients to ask doctors directly for more accurate information. This lack of coordination between nurses and doctors left patients feeling frustrated.

*In all honesty, I think … it was really a case that the staff really didn’t know anything about it. … They said, if you want anything, you have to talk to the Dr [name] who … who also works at a different hospital [hospital name], and so he would not be able to get there until maybe every other day. … So, there was a real issue with getting information from the nurses… it was quite frustrating for me—is just lack of information … they couldn’t add anything, any value to.*

(Patient 3)

#### 3.1.2. Nurses Emulating Hospital Ethos

Some patients expressed deep appreciation and admiration for the professionalism and extraordinary care provided by the nursing staff and did not think too much of their involvement in bedside handover. They found the nurses to be highly professional in their work. They believed that nursing staff met the highest standards of care and demonstrated a “genuine” sense of compassion and concern for their patients. Patients were amazed by the extent to which the nurses went to provide the best possible care. They expressed that hospital admissions could evoke fear in patients, particularly when grappling with health concerns or navigating the complexities of a post-surgery scenario with potential complications. What patients valued immensely was the compassion, kindness, and attentiveness displayed by the nurses, which contributed significantly to easing anxiety and instilling confidence in the nurses’ competence. This sense of reassurance not only fostered a feeling of safety but also translated into a positive and comforting experience through their hospital stay.

*Everybody was extremely professional. This is the highest standard and the nursing staff, are more genuinely caring. I think they go that extra mile in the caring department.*

(Patient 8)

*The compassion and the caring, unbelievable, they all bent over backwards to make sure that I was comfortable under my circumstance—post-surgery. I was in the best possible care. The overall experience, for me personally, was very positive given that hospitals are quite a scary environment to be in.*

(Patient 13)

### 3.2. Seeing the Value of Involvement in Bedside Handover

Even though patients may not have been involved in the bedside handover, they recognised its importance and acknowledged the potential value of being involved if given the opportunity. They believed that their active participation would not only enhance their understanding of their medical conditions but also empower them to contribute to their own care. They acknowledged that engaging in the handover discussion would foster a sense of collaboration and partnership with the healthcare team. Furthermore, they believed that their involvement would allow for personalised care plans that better address their individual needs and concerns. As a result, they would have experienced a greater sense of control and satisfaction in their healthcare journey.

#### 3.2.1. Desire to Know What’s Going on

Patients expressed a strong desire to be well-informed and reassured about their health status, treatment protocols, the subsequent steps in their care, and contingency plans in case of any unforeseen issues. Patients believed that having a clear understanding of their treatment journey would lessen anxiety and nervousness. Being involved in the nursing handover was a way for patients to find out what is going on with their treatment, making them feel “safe” and “confident” and ensuring they feel that they are “a part of their treatment” and “are not just a number on the chart”. They emphasised the importance of active involvement in their care, recognising it as a crucial aspect of their overall wellbeing.

*For the shift change, I think it’s important that patient could be a part of that conversation. … I think it’s also a comfort thing, like if you’re stuck in a hospital bed … the new nurse that you don’t know, [and] you’re gonna be relying on for the next shift. I found sometimes it was a little bit intimidating almost—that’s where my anxiety when you want to ask a question or you need help and things like that, [and] you don’t really know who your nurses are. … The more I got to know the nurses on the shift, … sort of having a proper handover where the patients involved makes that a little bit easier … makes you feel a bit more comfortable.*

(Patient 20)

*… If they were actually doing it [handover] face to face with you, it feels like you’re more … a part of your treatment. … It would be nice if it was like, you know, how are you feeling now? How have you gone over the last few hours? Just so the new nurse knows how you actually are feeling, and it makes you feel a little bit—not just a number on the chart, [and] feeling safer there. … Sometimes we’re not actually clear on what’s going on …, knowing that … they come in … talk … make you confident, … alert and competent … you feel a lot nicer being talked to rather than just being spoken about.*

(Patient 5)

#### 3.2.2. Contributing to Nursing Care

Patients embraced the opportunity to actively participate in their care and treatment through involvement in bedside handovers. Many believed that their participation could play a vital role in identifying and rectifying any inaccuracies, addressing concerns about medications, and contributing meaningfully to nursing practices, ultimately enhancing the quality of their care. They perceived that the collaborative approach empowered patients to take a more proactive role in their healthcare, fostering a sense of shared responsibility between healthcare providers and patients.

*In some instances, I thought I was contributing to their [nurse] work, when they promoted discussion with me. If I had known earlier about patient involvement, I will probably have shared more insights and concerns about my condition and treatment…*

(Patient 1)

*I think the benefit is that if a patient is involved, they can immediately communicate if they are experiencing any discomfort or excessive pain, or not feeling better than they should, or having adverse reactions to any medication… so they [healthcare team] can address these promptly and adjust the treatment plan accordingly. … Also, the best thing about having the patient being involved in my own handovers—would be eliminating errors. Well, it didn’t happen with me, but I can imagine from time to time the wrong paperwork gets dropped in and things like that.*

(Patient 11)

### 3.3. Barriers Hindering Patient and Family Involvement in Bedside Handover

Patients and families noted some barriers that impeded their involvement in bedside handover. These were sub-grouped into the organisational, nursing, and patient barriers.

#### 3.3.1. Organisational Barriers

***Informing patients of their involvement**: Most* patients said they did not know if involvement in the nursing handover was an option. They were unsure of their role in nursing handover or even whether they could ask questions. They often received routine inquiries from nurses about pain or medications as standard transactions where they felt nurses were in charge and patients just provided answers. Patients thought that formal information about their involvement could help them approach their interactions with nurses more thoughtfully. Informing patient involvement would have inclined patients to participate more actively and feel more at ease asking questions. Being informed that their participation in the handover was encouraged would have boosted their confidence in posing inquiries.

*No, I didn’t have any idea. No, no information at all about that I could or could not be [involve]. I certainly would have liked to participate, … [but] if no one tells you that you’re welcome to participate in the handover…*

(Patient 2)

*I think if it was clearly explained that that’s [patient involvement] the process and especially if they said why. Then I would be motivated and also feel confident … [and] possibly you would feel more inclined to participate.*

(Patient 6)

***Timing of handover**:* Bedside handovers were often conducted outside visiting hours, restricting the presence of patients’ families during this crucial time. Patients who were seriously ill, heavily medicated, or unconscious and consequently unable to comprehend or recall the ongoing proceedings expressed a desire for their families to be present during the handover. They conveyed that when their families inquired about the progress of their treatment, they were unable to provide accurate information. Recognising that family members could play a pivotal role in offering both physical and emotional support post-discharge, having them present during the handover was seen as advantageous. This way, patients believed, they could better understand the specifics of their follow-up care.

*No, no they [family] couldn’t, ’cause the [bedside] handover they did was mostly during the day and my partners … would be at work.*

(Patient 10)

*… I myself have been in situations where … I was suffering … something more of a significant injury and I was heavily medicated. … and any information had to be communicated to me. … [when] … my wife came in afterwards, … [asked] what did the doctor say? What’s happening? … and I just couldn’t remember. … And so, they worried. … I think that communication piece would be pretty important to immediate family that might have to step in and do things and stuff like that.*

(Patient 3)

Another patient also believed that involving a family member in the nursing handover was crucial, especially for older patients or those who were heavily medicated or those from non-English-speaking backgrounds. Without the presence of a family member, neither the patient nor their family could be involved in discussions about the treatment plan. This lack of knowledge about the medical condition could cause unnecessary stress for both patients and their families.

*… If people with second language was English and or more heavily medicated, or … older than me still again and you know, perhaps have some issues with their reception, so to speak. So that would be really, really hard, really difficult for the family, and that would just cause undue stress, [and] unnecessary trauma.*

(Patient 17)

Family members of patients shared similar sentiments, emphasising the importance of their involvement in the nursing bedside handover. They expressed that being part of the handover allowed them to identify and address any issues that needed attention. They wanted to know if the patient’s condition was improving or deteriorating. Not being informed about the patient’s condition and care could be very stressful for the family members.

*It would be good if the patient’s family could be involved in the handover. If that were available for that time,—they could understand what was going on as well. Uh, they could get an idea of what the problem is. You know if it’s going to be fixed soon, so to speak or corrected, or if there’s anything that the patient goes home with that should be attended to looked after. They should be involved in all of that sort of thing where possible. They would have to rely on what the patient interpretation of the knowledge.*

(Patient’s husband 12)

*I probably wasn’t there at the time when it [handover] occurred. No, specific effort or arrangement was made for me to be present at a handover. It was very important for me to know exactly what my wife was going through and what treatment she was receiving. Also, if her condition was improving or not it, or deteriorating more. it’s sort of gives you a little bit of an insight onto how things are going. … So, makes you feel a little bit more at ease. Not knowing what is happening, you get very depressed and very sad.*

(Patient’s husband 23)

Family members of another two patients who had dementia were concerned that they often couldn’t recall what the nurses talked about or detail of their treatment plan. It was particularly important because they were responsible for the patient’s care after hospital discharge.

*My wife for almost 90–95% would be unable to recall if the two nurses discussed the handover in her presence, any of the discussion that they had. … I would visit her after, … and I would say, how things going? … have the nurses said anything? or giving you any advice? do you know when you’re going to be discharged? what medication are you being given? It would have been much more beneficial if, if I’d been present and being able to take it in and understand what had been done and what needed to be done when she was discharged.*

(Patient’s husband 24)

*… There’s one thing that he does tend to forget, and so, a lot of time, when I come in to see him and I asked him ‘what’s going on’ and he can’t remember…*

(Patient’s wife 7)

#### 3.3.2. Nursing Barriers

***Not at bedside**:* Patients mentioned that nurses often conducted their handovers either on the other side of the curtains or in the doorway, where patients could overhear the discussions but could not participate as they were not nearby.

*Well, I suppose the fact that they didn’t come in. … You could hear them doing that. I was in a room where it was a four-person room. And yeah, sometimes you would hear them and transferring information. … sometimes then after they’ve done that, they would come and see you pretty soon afterwards, but sometimes they didn’t come and see you until it was time. … half the time this happened when the curtains were closed, and they would be the other side of the curtain. So, you couldn’t see them, you could just hear them discussing you. I think it would be better if they, when they were doing that, if they came in next to your bed. … It would be nice to have participated better because when they’re outside the curtain you couldn’t saw, shout and ask questions. I mean, I would have had to have got out of bed, got up and spoken to them, or if they came in and were next to me. I could. Then it would then be easier to ask questions.*

(Patient 14)

*Always in the doorway. If they’re not really in the room, how can you participate? There was rarely that I got to hand over in the room. … I’d be lying down or sitting up and there’d be a new face like that—I am not being introduced to them or anything like that. … and they weren’t in the room.*

(Patient 15)

Patients expressed a preference for nurses to discuss their care in their presence rather than in the hallway or on the other side of a curtain. Patients felt that it was unprofessional for the nurses to conduct handovers in the hallway, which could potentially breach their privacy and confidentiality. However, they did not share the same level of apprehension when the handover occurred at the bedside in a shared room, where other patients and visitors might overhear sensitive information. They acknowledged that ensuring patient confidentiality in a hospital setting, especially in shared wards, was challenging. Despite efforts to respect privacy, the nature of shared wards meant that patients inevitably heard doctors discussing others’ medical conditions during rounds. Yet, they thought that the hospital was a private place.

*… If I could hear what they were saying about the people in the room next door to me, then probably the people in the room next door to me could hear what they were saying about me. … It just wasn’t anything to really be overly concerned about, except that it’s not really good for them to have those sorts of discussions where other people can hear, you know. … it wasn’t violet speech, but it was a sort of a partial effort to not talk too loud … so, it just did come across as being unprofessional.… I just would have been quite comfortable coming in at that discussion in front of me so I could hear so well. It’s like, what’s the secret? It’s about my care … [so] come in and share because there’s something going on I’d like to know about it, … just have that discussion here.*

(Patient 18)

*When you’re in a shared room, there is really no privacy, because the curtain doesn’t block the sound, so everyone else in that room knows exactly what’s going on. But I don’t think it matters whether it’s on one side of the curtain or the other. Everyone else in that room is gonna hear anyway. I feel like a little bit private, but you know the person next to you that you have all heard how many times you’ve gone to the toilet. So, I hear that whether they’re doing outdoor or just next to the curtain, people will hear it anyway, but [I] prefer they do the same thing next to my bed.*

(Patient 20)

Patients believed that while patient information should be respected in a hospital setting, there should not be unnecessary secrecy, and medical staff could perform their duties transparently. Their preference remained for the handover to take place in front of them with their active involvement.

*Confidentiality, no, I didn’t worry about that, I don’t worry about confidentiality, not on when you’re in the hospital, especially not in visiting hours. It’s like a dignity, … and in a hospital that the staff, nurses and doctors have to do what they have to do.*

(Patient 19)

*The nature of the hospital is such that confidentiality is actually really, really hard, particularly in a shared ward. Nobody wants to hear other people’s issues, but you can’t help but hear them. … you’re hearing the doctors come around on their rounds. You know, you’re hearing what they’re saying to the person next door. But here’s one of the more private places.*

(Patient 22)

***No explicit invitation**:* In certain instances, when nurses conducted handovers at the patient’s bedside, there was a lack of invitation or initiative to engage the patients in the process. While some patients believed they did not need to know every detail, others found it intriguing that they were not actively engaged in the process.

*Sometimes they would introduce saying that they’re doing a little handover. Sometimes they’ll just hand over. Sometimes they’ll include you in it like they’ll just ask you to clarify some stuff. Other times they won’t. They’ll just rush through it or something like that.*

(Patient 21)

*I’m not saying I feel like they’ve neglected me. It’s not like that at all. I just think that there could have been a little bit more interaction, Nice to have somebody come in and just say “hello, how are you”,—not coming in to have a chat. I didn’t need this … but they didn’t engage me in conversation, and I just sort of I had my own. If you hadn’t gotten in the room and you hadn’t gotten them engaging with you, then how are you going to be involved in the handover? You’re not gonna be involved, and I don’t think I was involved in it. … Just one nurse came and said, well, I’m your new nurse. And then she checked and then disappeared.*

(Patient 16)

One family member also recounted a similar situation, expressing the belief that there might not have been much for her husband to be involved in:

*… During those times, it’s really nothing much to say for [husband’s name], like for his pain. … [Nurses were] informing him … “a new nurse taking over for you” like that sort of thing, and then they say “alright, we’re just out the door” and they’re doing all the paperwork, and [husband’s name] is like ‘yeah, no worries, that’s fine’. Yeah, so that’s basically how they did.*

(Patient’s wife 7)

#### 3.3.3. Patient Barriers

***Fear of asking questions**:* The fear of asking nurses questions emerged as another barrier that prevented patients from actively participating in nursing bedside handovers. Patients worried that if they posed too many questions, nurses might perceive them negatively, and therefore, they did not want to be labelled as a “troublemaker” or “talkative person”. Patients, therefore, often tried to maintain a polite demeanour. They believed that only the “noisy” or “difficult” patients, or those with “hypochondriac tendencies” or “addictive personality”, tended to ask excessive questions and exaggerate their situations. Acknowledging the nurse’s hard work, some patients felt a sense of guilt when making enquiries and made a conscious effort not be perceived as a “nuisance”.

*… I certainly would have liked to participate, … [but] I didn’t ask that many questions, you don’t want to be more trouble than you need to be. you know, they were working hard.*

(Patient 10)

*I thought, almost so, what are those people that doesn’t like to be a lot of trouble? And I feel kind of guilty because I know they’re always busy … on the ward and they work pretty hard, and they were all working double shifts.*

(Patient 3)

**Patient’s condition:** Patients’ physical and mental conditions further restricted their involvement in nursing bedside handovers. When they were unwell or wanted to sleep, they preferred not to participate. Additionally, some perceived the nurse as ‘the boss,’ retaining the decision-making role and leaving patients feeling excluded from the process.

*For that hand over, I felt very confident that all of my needs and … medical needs were being met. Sometimes … if you’re asleep or you’re not feeling well or something like that and handover happens, then you obviously not gonna be involved—Let them sleep. It’s very hard to get sleep in that place, so once they’re sleep, let them sleep.*

(Patient 4)

*… They [nurses] are the boss, I do not care if patients are involved or not. … I was content, I wasn’t worried. I wasn’t offended. Nothing. I was quite happy with what happened.*

(Patient 9)

## 4. Discussion

The impact of this study is significant due to its comprehensive exploration of patients’ and families’ perceptions regarding their involvement in nursing bedside handovers in both private and public hospitals, enhancing the broader applicability of its findings within the context of the Australian healthcare setting. The use of a descriptive qualitative approach with in-depth and semi-structured interviews offers detailed and nuanced descriptions of participant experiences of their involvement in the nursing bedside handover process. Conducting bedside handover within a patient-and-family-centred care framework has been shown to reduce patient safety incidents and enhance patient satisfaction [5]. The Standard Operating Protocol for Implementing Bedside Handover in Nursing [23] recommends that both patients and families be formally invited to participate in nursing bedside handovers. Before any bedside handovers, nurses should greet and formally invite patients and their families to participate. Nurses are also advised to inquire whether patients and their families have questions or comments and to confirm and clarify information during the handover process. The study findings echo the known challenges of implementing a person-centred care approach [24], which is also conceptualised as patient-and-family-centred care [25]. Patient-centred care emphasises treating each individual with respect and recognising their unique human identity rather than reducing them to a medical condition [26]. Its implementation in healthcare settings involves actively understanding the priorities of patients, their families, carers, and support people to establish trust and mutual respect [27].

From the findings, we can infer that health services within this study were striving to implement a patient-care approach through bedside handovers. In particular, participants, through their multiple hospital admissions, observed structural improvements in nursing handover processes. These improvements were evident in heightened nurse professionalism, better communication, and consistent practices that ensured attentive care during their hospital stays. In this study, patients were satisfied with nursing care and the professional conduct of nurses. Patients stressed that active involvement in their care was vital for their overall wellbeing. In addition, they considered family members’ involvement as also being crucial, particularly when the patients were too unwell to understand anything the nurse told them during the bedside handover. Yet, the findings of this study reveal that patient and family active involvement was largely absent during bedside handover. The findings of this qualitative investigation confirm the results from the cross-sectional survey conducted with a broader participant cohort as part of our larger study [15].

Several factors hindered patients’ and families’ involvement in bedside handover. Both the cross-section surveys [15] and in-depth interviews in the current study revealed that handovers often occurred away from the patient’s bedside. When scheduled at the bedside but outside visiting hours, family involvement was limited. Other studies also reported the absence of beside handover [28,29], despite patients’ preference for handover at the bedside to facilitate their participation [30]. There has not been much research on how patients participate during different nursing shift changes. It is important to study this because shift changes can happen at different times in different settings. Understanding patient involvement during these times and providing advance notice of handover timings can help improve patient and family involvement [1].

Maintaining patient privacy and confidentiality is complex and critical during bedside handover, especially in shared rooms and during visiting hours. Participants in our study acknowledged the challenges of maintaining privacy and confidentiality in hospitals, particularly in shared rooms, but were not “overly concerned” and preferred handovers next to their bed. However, both quantitative [15] and qualitative results indicated participants’ concerns about privacy breaches when handovers were conducted in hallways or outside the curtain of hospital beds. Anshasi and Almayasi [12], in their systematic review, found breaches of confidentiality and privacy violations to be significant barriers to bedside handover, emphasising the importance of considering patients’ preferences when maintaining confidentiality during bedside handover. Similarly, Malfait et al. [31] pointed out that privacy and confidentiality should not justify moving bedside handovers outdoors. If privacy issues arise, healthcare providers should implement appropriate measures to safeguard patients during bedside handover.

In this study, participants were uncertain if they were allowed to actively participate in bedside handovers or the extent of their involvement in discussing their treatment progress. This uncertainty was consistent with findings from a cross-sectional survey [15], where patients felt they had limited opportunities to discuss difficult clinical situations with the nurses or share their opinions and concerns. Similarly, Rifai et al. [29] reported that patients often lacked understanding of bedside handovers and their role in them, hindering their involvement. Engaging patients in their care is challenging due to the ambiguous nature of involvement, with many patients feeling excluded [32].

Both the cross-sectional survey [15] and qualitative descriptive study revealed that not all patients desired involvement. Factors such as fear of asking questions and lack of energy and initiative significantly contributed to their non-involvement. This finding aligned with Unbeck et al. [33], who found that patients’ lack of experience in healthcare contexts hindered their ability to take initiative and understand their expected level of involvement in elective surgical procedures. This lack of awareness and experience affected patient motivation, leading them to adopt a more passive role and view certain tasks as the nurse’s responsibilities [29]. Therefore, fostering meaningful relationships between patients and nurses are recommended to address individual needs and preferences regarding involvement [33]. Additionally, clearly explaining the importance of active participation in their own care to patients and families can enhance their involvement [33].

### 4.1. Relevance to Clinical Practice

Bedside handover that includes patient and family involvement in their care progress is still an evolving practice. Meaningfully engaging patients in collaborative care planning requires a unique set of skills. This study marks the inaugural exploration of patient and family involvement in nursing bedside handovers in WA. It stands among a limited number of investigations on this subject within the research field. This study offers valuable insights into the challenges and factors which hinder patients and their families’ involvement in bedside handover. Additionally, it can be a valuable resource for the two hospitals under study as well as the healthcare sector in WA and Australia at large, contributing to the development of practical guidelines and effective implementation plans to elevate nursing care practices involving patients and their families in bedside handover.

### 4.2. Limitations

Due to resource limitations, the interviews were conducted in English, which may have excluded patients and families from diverse cultural and linguistic backgrounds. The invitation to participate in this study was extended to all inpatient wards and units, except intensive care, coronary care, same-day care units, and emergency departments in both hospitals. However, we did not analyse data according to specific wards or units during their participation. Variations in ward-level nursing communication and culture could impact patient and family experiences. This study was conducted in only two hospitals and relied on participants’ personal experiences, which may limit the transferability of the findings. Additionally, the low number of family members who participated in this study may also impede the wider application of the results. While there was a low number of family participants, the data they provided clearly indicates further work is required to engage family members in bedside handover.

## 5. Conclusions

In conclusion, this study found three major themes related to patients’ and families’ perceptions and experiences of their involvement in nursing bedside handover. The results showed that participants recognised the importance of involving patients and their families in nursing bedside handover. The findings also revealed that despite being acknowledged as best practice, the routine inclusion of patients and their families in bedside handover was often inconsistent and varied in its degree of implementation. The study identified various challenges and barriers to optimal patient and family involvement across multiple levels—organisational, staff, and patient. These included issues such as informing patients of their right to participate, the timing of handover, absence of bedside handover, lack of invitation for patient and family involvement, patient’s fear of asking questions, and patient personality or health condition. To enhance patient and family involvement, targeted strategies and training programs are recommended for nursing staff to reinforce the benefits of patient and family involvement and develop skills in patient engagement and communication. Additionally, a periodic review of bedside handover procedures is necessary to determine the extent of patient and family involvement and staff adherence to handover policies and procedures. Further research into patient and family involvement in nursing bedside handover in other hospital settings is also recommended to build a more comprehensive picture of these critical domains of patient-and-family-centred care.

## Figures and Tables

**Table 1:** Characteristics of the study participants.

Characteristics	Number of Participants (n = 24) n (%)
*Gender*	
Female	13 (54%)
Male	11 (46%)
*Age*	
20–29	1 (4%)
30–39	3 (13%)
40–49	3 (13%)
50–59	4 (17%)
60–69	4 (17%)
70–79	6 (25%)
80–89	3 (13%)
*Relationship*	
Patient	20 (83%)
Family	4 (17%)
*Country of birth*	
Australia	13 (54%)
England	7 (29%)
Italy	1 (4%)
Malaysia	2 (8%)
Papua New Guinea	1 (4%)
*Level of education*	
Higher Secondary School or below (Yr 12)	6 (25%)
Graduate	8 (33%)
Postgraduate	3 (13%)
Trade/TAFE	7 (29%)
*Employment status*	
Full-time	7 (29%)
Part-time/casual	2 (8%)
Retired	12 (50%)
Unemployed	3 (13%)
*Admitted to any of the two hospitals in the last 5–7 years*	
1–3 times	14 (58%)
4–6 times	3 (13%)
7–9 times	5 (21%)
10 times and more	2 (8%)
*How many nights stayed in the current admission*	
1–3 nights	9 (38%)
4–6 nights	5 (21%)
7–9 nights	4 (17%)
10 nights and more	6 (25%)

## Data Availability

The original contributions presented in this study are included in this article. Further inquiries can be directed to the corresponding author.

## Supplementary Materials

The following supporting information can be downloaded at https://www.mdpi.com/article/10.3390/nursrep15020051/s1: the Standards for Reporting Qualitative Research (SRQR) Framework checklist.

## Public Involvement Statement

There was no public involvement in any aspect of this study.

## Guidelines and Standards Statement

The study was guided by the Standards for Reporting Qualitative Research (SRQR) Framework.

## References

[1] Tobiano G., Bucknall T., Sladdin I., Whitty J.A., Chaboyer W. (2018). Patient participation in nursing bedside handover: A systematic mixed-methods review. Int. J. Nurs. Stud..

[2] Wiklund I., Sahar Z., Papadopolou M., Löfgren M. (2020). Parental experience of bedside handover during childbirth: A qualitative interview study. Sex. Reprod. Healthc..

[3] Chaboyer W., McMurray A., Wallis M., Chang H. (2008). Standard operating protocol for implementing bedside handover in nursing. J. Nurs. Manag..

[4] ACSQHC (2021). Australian Commission on Safety and Quality in Health Care. National Safety and Quality Health Services Standards.

[5] Ismuntania K., Nursalam F.R., Nurlela Mufida I., Maulina Iriyanti Z., Muzaffar S.D., Fitri Apriani A.S. (2023). Bedside Handover using Patient Family Centered Care on Patient Safety and Patient Satisfaction: A Systematic Review. J. Posit. Psychol. Wellbeing.

[6] Dellafiore F., Arrigoni C., Grugnetti A.M., Zaffino G., Calorenne V., Pitella F., Rosa D., Caruso R. (2019). Bedside nursing handover and organisational will to achieve personalisation within an Italian Cardiac SurgeryUnit: The nurses’ viewpoint through a qualitative study. Prof. Inferm..

[7] Jimmerson J., Wright P., Cowan P.A., King-Jones T., Beverly C.J., Curran G. (2021). Bedside shift report: Nurses opinions based on their experiences. Nurs. Open.

[8] Hada A., Jack L., Coyer F. (2019). Using a knowledge translation framework to identify barriers and supports to effective nursing handover: A focus group study. Heliyon.

[9] McCloskey R.M., Furlong K.E., Hansen L. (2019). Patient, family and nurse experiences with patient presence during handovers in acute care hospital settings: A systematic review of qualitative evidence. JBI Evid. Synth..

[10] DeCelie I. (2020). Patient participation strategies: The nursing bedside handover. Patient Exp. J..

[11] Mullen A., Isobel S., Flanagan K., Harman K. (2021). Involving mental health consumers in nursing handover: A qualitative study of consumer perspectives. Issues Ment. Health Nurs..

[12] Anshasi H., Almayasi Z.A. (2024). Perceptions of Patients and Nurses about Bedside Nursing Handover: A Qualitative Systematic Review and Meta-Synthesis. Nurs. Res. Pract..

[13] Bressan V., Cadorin L., Stevanin S., Palese A. (2019). Patients experiences of bedside handover: Findings from a meta-synthesis. Scand. J. Caring Sci..

[14] Creswell J.W., Creswell J.D. (2017). Research Design: Qualitative, Quantitative, and Mixed Methods Approaches.

[15] Ghosh M., Nosaka K., Saunder R., Gallagher O., Towell-Barnard A., Ghosh D., Gent L., Coventry L. (2024). Patient perception of involvement in nursing bedside handover: A cross-sectional study. JAN.

[16] Sandelowski M. (2010). What’s in a name? Qualitative description revisited. Res. Nurs. Health.

[17] Doyle L., McCabe C., Keogh B., Brady A., McCann M. (2020). An overview of the qualitative descriptive design within nursing research. J. Res. Nurs..

[18] Bradshaw C., Atkinson S., Doody O. (2017). Employing a qualitative description approach in health care research. Glob. Qual. Nurs. Res..

[19] O’Brien B.C., Harris I.B., Beckman T.J., Reed D.A., Cook D.A. (2014). Standards for reporting qualitative research: A synthesis of recommendations. Acad. Med..

[20] Kiger M.E., Varpio L. (2020). Thematic analysis of qualitative data: AMEE Guide No. 131. Med. Teach..

[21] Krefting L. (1991). Rigor in qualitative research: The assessment of trustworthiness. Am. J. Occup. Ther..

[22] NHMRC (2023). The National Statement on Ethical Conduct in Human Research.

[23] Chien L.J., Slade D., Dahm M.R., Brady B., Roberts E., Goncharov L., Taylor J., Eggins S., Thornton A. (2022). Improving patient-centred care through a tailored intervention addressing nursing clinical handover communication in its organizational and cultural context. J. Adv. Nurs..

[24] van Belle E., Giesen J., Conroy T., van Mierlo M., Vermeulen H., Huisman-de Waal G., Heinen M. (2020). Exploring person-centred fundamental nursing care in hospital wards: A multi-site ethnography. J. Clin. Nurs..

[25] Franck L.S., O’Brien K. (2019). The evolution of family-centered care: From supporting parent-delivered interventions to a model of family integrated care. Birth Defects Res..

[26] McCormack B., McCance T., McCormack B., Klopper H. (2017). Person-Centred Practice in Nursing and Health Care: Theory and Practice.

[27] Johnsson A., Wagman P., Boman Å., Pennbrant S. (2018). What are they talking about? Content of the communication exchanges between nurses, patients and relatives in a department of medicine for older people-An ethnographic study. J. Clin. Nurs..

[28] Street M., Dempster J., Berry D., Gray E., Mapes J., Liskaser R., Papageorgiou S., Considine J. (2022). Enhancing active patient participation in nursing handover: A mixed methods study. J. Clin. Nurs..

[29] Rifai A., Afandi A.T., Hasanah A. (2020). Bedside Nursing Handover: Patient’s Perspective. NurseLine J..

[30] Oxelmark L., Whitty J.A., Ulin K., Chaboyer W., Gonçalves A.S.O., Ringdal M. (2020). Patients prefer clinical handover at the bedside; nurses do not: Evidence from a discrete choice experiment. Int. J. Nurs. Stud..

[31] Malfait S., Van Hecke A., Van Biesen W., Eeckloo K. (2019). Is privacy a problem during bedside handovers? A practice-oriented discussion paper. Nurs. Ethics.

[32] Nilsson M., From I., Lindwall L. (2019). The significance of patient participation in nursing care—A concept analysis. Scand. J. Caring Sci..

[33] Unbeck M., Lidgren F., Tabbakh E., Nymark C. (2023). The patient’s experience of participation when admitted for elective surgical procedures: An interview study. Int. J. Qual. Stud. Health Well-Being.

