# Supplementary material for: Patient and Family Involvement in Nursing Bedside Handover: A Qualitative Descriptive Study of Consumer Perceptions of Nursing Care
Source: Nurs Rep. 2025 Feb 3;15(2):51. doi: 10.3390/nursrep15020051 (PMC11858350; doi:10.3390/nursrep15020051)
Supplement: Supplementary file 1 [file nursrep-15-00051-s001.zip › nursrep-3365688-supplementary.pdf]

Standards for Reporting Qualitative Research (SRQR) checklist.

No	Item	Page
Title		
S1	Concise description of the nature and topic of the study identifying the study as qualitative or indicating the approach (e.g. ethnography, grounded theory) or data collection methods (e.g. interviews, focus group)	1
Abstract		
S2	Summary of key elements of the study using the abstract format of the intended publication; typically includes background purpose, methods, results and conclusions.	P.1
Introduction		
S3	Problem Formulation: Describes the problem or phenomenon of interest.	P. 2-3
S4	Purpose or Research Question: Clearly states the research questions or objectives.	P. 3
Method		
S5	Research Design: Describes the qualitative approach and research design (e.g., grounded theory, ethnography).	P. 3-4
S6	Participant Selection: Describes the process and criteria for selecting participants.	P. 4-5
S7	Setting: Describes the setting and context in which the research was conducted.	P. 4
S8	Data Collection: Describes the methods and procedures for data collection.	P. 5
S9	Data Analysis: Describes the process and methods for analyzing the data.	P. 5-6
S10	Researcher Characteristics and Reflexivity: Describes the researcher's characteristics, background, and potential biases.	P. 6
S11	Ethical Considerations: Describes ethical issues and how they were addressed, including informed consent and confidentiality.	P. 6
Results		
S12	Findings: Presents the findings of the study, organized around key themes or categories.	P. 7; Table- P 7-8, Figure 1, P-9
S13	Data: Provides rich, thick descriptions of the data to support the findings.	P. 10
S14	Context: Describes the context and setting of the data.	P. 10-21
S15	Participant Quotations: Provides direct quotations from participants to illustrate key points.	P. 10-21
Discussion		
S16	Interpretation: Interprets the findings in relation to the research questions and existing literature.	P. 21-24
S17	Implications: Discusses the implications of the findings for practice, policy, and future research.	P. 24
S18	Limitations: Acknowledges the limitations of the study.	P. 24-25
Conclusion		
S19	Summary: Provides a concise summary of the study and its findings.	P. 25
S20	Recommendations: Offers recommendations based on the study's findings.	P. 25
Supplementary Information		
S21	Appendices: Includes supplementary materials such as interview guides, data collection tools, or additional data.	Supplementary document 1
